# Gaps in Support for Two Autistic Individuals in Adulthood: A Case Report

**DOI:** 10.7759/cureus.116482

**Published:** 2026-09-18

**Authors:** Alfredo de Jesús Garza Guerra, Elisa del Rocio Mata Cortes, Gabriela Hilian Adame Rocha, Stefan Mauricio Fernández Zambrano

**Affiliations:** 1 Psychiatry, Hospital Universitario Dr. José Eleuterio Gonzalez, Monterrey, MEX; 2 Psychiatry, Jalisco Institute of Mental Health and Addictions, Universidad de Guadalajara, Jalisco, MEX

**Keywords:** adulthood, autism, continuity of support, social participation, support gaps, transition to adulthood

## Abstract

The transition to adulthood can leave autistic people without the educational, therapeutic, social, and community supports available earlier in life. Adults identified as autistic later in life may encounter a different but related gap, receiving a meaningful explanation for longstanding experiences without obtaining the practical support needed to pursue education, employment, relationships, or community participation. This report describes two autistic individuals whose different trajectories converged in a shared loss of continuity.

A 19-year-old woman, identified as autistic in childhood, lost access to her special education school and autism-focused services after reaching their age limits. Approximately six months later, she developed depressive symptoms, anxiety, irritability, and reduced motivation. These concerns remitted with psychiatric treatment, but appropriate adult services remained unavailable. She subsequently enrolled in an online upper-secondary education program with daily assistance from her mother.

A 26-year-old man withdrew from university after becoming increasingly overwhelmed by academic, organizational, and social demands. Following a period of depression, anxiety, reduced activity, and increasing social withdrawal, he underwent several mental health evaluations before being identified as autistic in adulthood. His emotional and functional status subsequently improved, but no formal pathway was available to support educational re-entry or provide individualized adult support. An opportunity provided through his church community facilitated his return to employment, where he had remained for approximately two years at the time of this report.

These cases illustrate different pathways to the same problem: the loss or absence of coordinated support in adulthood. Clinical improvement or autism identification alone does not ensure access to education, employment, relationships, community participation, or greater autonomy. Adult support should therefore be individualized, responsive to changing needs, and oriented toward participation and autonomy rather than dependence. Support need not rely exclusively on healthcare services and may involve coordinated contributions from educational, employment, family, social, and community resources.

## Introduction

Autism represents a lifelong variation in human neurodevelopment characterized by differences in social communication and interaction, alongside restricted or repetitive patterns of behavior, interests, or activities, which may include distinctive sensory experiences. These characteristics vary considerably among autistic people and may be associated with different strengths, challenges, and support needs across the life course [1]. Depending on individual needs and preferences, formal supports may include educational accommodations, speech and communication support, occupational therapy, mental healthcare, assistance with daily living, vocational services, and programs that facilitate social and community participation [2].

Despite the lifelong nature of autism, formal services and specialized programs remain disproportionately concentrated in childhood and adolescence [2,3]. Entering adulthood does not end the need for support; rather, support needs may extend across education, employment, healthcare, relationships, community participation, and increasing autonomy [2,4]. However, continuity across these areas is not always maintained during the transition to adulthood [3,4].

The reduction in services surrounding adulthood has been described as a 'service cliff' [3,4]. Longitudinal studies indicate that this decline may begin before high school exit and become more pronounced afterward [3]. In one study, nearly 40% of autistic young adults in the study population received none of the assessed health and support services after leaving high school [5]. Beyond changes in service access, the transition to adulthood is also associated with challenges in education and employment, with autistic young adults experiencing barriers to participation in postsecondary education and paid employment [6,7].

The consequences of this discontinuity extend beyond service utilization. The loss of structured routines, familiar relationships, educational opportunities, therapeutic supports, and meaningful community activities may contribute to social isolation, reduced participation, and emotional distress [8]. These challenges are particularly relevant given the high rates of co-occurring mental health conditions, including depression and anxiety, among autistic people [9]. However, well-being should not be understood solely through the lens of individual psychiatric difficulties, as it is also shaped by environmental accessibility, opportunities for participation, and the availability of appropriate support [2,10]. In this context, access to community settings or activities does not necessarily translate into meaningful inclusion. Environmental, interpersonal, and support-related barriers may continue to limit participation and engagement in community life [10]. Consequently, clinical improvement or greater access to community resources may address some barriers without necessarily ensuring sustained participation, supportive relationships, or opportunities for the future [10].

A comparable but distinct gap may affect people who are first identified as autistic in adulthood. Adult identification can provide a meaningful framework for understanding longstanding social, communication, sensory, and organizational experiences, allowing individuals to reinterpret earlier difficulties within a more coherent developmental context [11,12]. However, identification alone does not necessarily translate into access to appropriate support across education, employment, mental health, social participation, and future planning. Research on post-identification experiences indicates that autistic adults frequently describe available support as fragmented, insufficient, or poorly aligned with their longer-term needs [12,13]. Accordingly, autistic adults and their support persons have emphasized the importance of individualized, accessible, and practical assistance that extends beyond the provision of general information [14].

This report presents two autistic individuals whose different trajectories converged in a shared loss of continuity. One received structured educational, therapeutic, and community support during childhood and adolescence but lost access to these services after reaching their age limits. The other remained unidentified as autistic throughout childhood and adolescence and was identified only in adulthood, after difficulties at university and previous mental health evaluations. Although both the decline in services during the transition to adulthood and gaps in post-identification support have been described in the literature, important gaps remain between recognizing these problems and providing coordinated, practical support in adult life.

By presenting these cases side by side, this report aims to illustrate three interconnected challenges: (I) the loss of established support during the transition to adulthood, (II) the consequences of autism remaining unidentified until adulthood, and (III) the gap between clinical recognition or improvement in co-occurring mental health symptoms and meaningful participation in education, employment, relationships, and community life. The cases therefore highlight not only where continuity may be disrupted but also how different developmental and clinical pathways can leave autistic adults and their families responsible for constructing their own solutions when formal systems do not provide a clear pathway forward.

## Case presentation

The principal clinical, educational, and support-related events in both cases are summarized in Table 1. The timeline highlights the sequence from developmental and educational history through major transition-related events, the emergence of mental health concerns, treatment, clinical improvement, and subsequent efforts to re-engage in education, employment, and community life.

**Table 1:** Clinical, educational, and support-related timeline of the two cases Ages and time intervals are approximate where indicated (with '~') and are based on the clinical histories available for this report. The timeline presents documented events and does not imply a causal relationship between educational or support-related transitions and the subsequent emergence of mental health concerns.

Stage/event	Case #1	Case #2
Developmental history	Identified as autistic at age five through clinical and neuropsychological assessment; borderline intellectual functioning and communication support needs.	No reported significant developmental delays or major developmental concerns; longstanding preference for interactions with one or two familiar people and a brief, direct, and concrete communication style.
Childhood and adolescent education/support	From age eight, attended special education and nonprofit autism-focused foundations providing therapeutic, social, and recreational activities.	Good academic performance throughout primary and secondary education; generally interacted with one or two familiar peers.
Major transition	~Age 17: Completed secondary school and lost access to school-based and autism-focused services because of age restrictions; no transition plan or equivalent adult services were available.	Entered university but became increasingly overwhelmed by academic, organizational, and social demands; withdrew after one semester.
Mental health concerns	~Six months after supports ended: persistent low mood, reduced motivation, anxiety, irritability, and increased appetite.	Following university withdrawal, there was substantial reduction in daily structure and activities, increasing social withdrawal, depressive and anxiety symptoms, and reduced motivation and participation in previous activities.
Initial mental health care	~Age 18: Sought psychiatric care accompanied by her mother.	Within ~six months of university withdrawal, he began psychotherapy, which continued for two years with partial improvement. He underwent two clinical evaluations before subsequent reassessment by our team.
Psychiatric treatment	Treatment was initiated with sertraline and aripiprazole, with the latter used to address irritability. Dosages were gradually adjusted according to clinical response and tolerability, reaching sertraline 100 mg/day and aripiprazole 10 mg/day.	Fluoxetine 20 mg/day and clonazepam 0.5 mg/day for two years. Following his second clinical evaluation, olanzapine 10 mg/day was added and continued for approximately the final year after concern was raised for a possible prodromal phase of schizophrenia.
Reassessment	Continued psychiatric follow-up, with treatment adjusted according to clinical response and tolerability.	Reassessed by our team, with specific evaluation of the previously raised concern regarding possible psychotic symptoms. Clinical assessment did not identify clinically significant psychotic features, and developmental history indicated that his social-communication characteristics had been present since childhood and preceded the depressive episode. Clinical and psychometric assessment supported identification as autistic in adulthood.
Treatment modification	Sertraline 100 mg/day and aripiprazole 10 mg/day were maintained after clinical stabilization.	Fluoxetine, clonazepam, and olanzapine were gradually discontinued, and treatment was transitioned to venlafaxine 75 mg/day and risperidone 1 mg/day. Risperidone was subsequently discontinued, while venlafaxine 75 mg/day was maintained.
Clinical outcome	~One-year follow-up: Depressive symptoms, anxiety, and irritability had remitted. No clinically significant adverse effects or weight changes were observed, and both medications were continued at the same dosages.	~Six months after treatment modification, he returned to a level of daily functioning similar to that before the onset of his depressive symptoms. At one-year follow-up, he remained emotionally stable, with sustained remission of depressive and anxiety symptoms. No psychotic symptoms emerged during follow-up.
Current education/employment	Age 19: After alternative educational options were explored collaboratively with her and her family, she enrolled in a formal online upper-secondary education program completed from home, with daily assistance from her mother.	Age 26: Employed within his church community for ~two years. With greater clinical and functional stability, educational alternatives were explored collaboratively with him and his family.
Current goals/remaining support gap	Wishes to pursue a university degree and have a romantic partner. Although online education enabled her to resume formal studies, suitable adult educational and community supports remain limited.	Considering resuming university studies through an online program; at the time of this report, no formal pathway for educational re-entry or individualized adult support had been established.

Case #1 featuring the end of support at the threshold of adulthood

A 19-year-old autistic woman had been identified as autistic at the age of five through a comprehensive evaluation that included clinical and neuropsychological assessment. She had borderline intellectual functioning and required support with language and communication. She communicated verbally and could participate in meaningful conversations when given sufficient time and patience. Some words were difficult to understand because of differences in pronunciation, and she occasionally reversed pronouns. As the present report focuses on the discontinuity of support during the transition to adulthood rather than on diagnostic or differential diagnostic considerations, a detailed reconstruction of her early developmental history was beyond its scope. Written informed consent for publication of this case report was obtained from the individual and her mother. She independently completed basic activities of daily living. Activities outside the home generally occurred with family accompaniment, primarily because of concerns about her safety.

At the age of eight, she began attending a special education school and participating in nonprofit foundations providing autism-related therapeutic, social, and recreational activities. At school, educational support was adapted to her learning pace and abilities, while the community organizations provided structured therapeutic and social activities. Together, these settings offered a predictable routine, individualized support, and regular opportunities to interact with others. Although she formed relationships within these structured activities, those relationships rarely continued beyond the programs.

At approximately 17 years of age, after completing secondary school, she lost access to both her school and the autism-focused organizations because of age restrictions. No transition plan was developed, and no equivalent adult services were available. Her mother subsequently searched for educational and community programs but was unable to identify services appropriate to her daughter’s communication, social, and learning needs.

Approximately six months after these supports ended, she developed persistent low mood, reduced motivation, anxiety related to the loss of her routine, irritability during interactions with her parents, and increased appetite. She did not report suicidal thoughts and had no self-injurious or aggressive behavior. Approximately one year after leaving these services, at around 18 years of age, she and her mother sought psychiatric care.

A mental status examination and an assessment of depressive symptoms were conducted, with information obtained directly from the patient and supplemented by her mother when appropriate. The assessment was adapted to her communication and cognitive profile rather than relying exclusively on standardized self-report measures. Weight and vital signs were within clinically acceptable ranges and showed no meaningful change from her previous baseline. Physical and neurological examinations were unremarkable, and routine laboratory investigations showed no clinically significant findings. No radiological investigations were considered clinically indicated.

Treatment was initiated with sertraline and aripiprazole, with the latter used to address irritability. Medication dosages were gradually adjusted according to clinical response and tolerability, reaching sertraline 100 mg once daily and aripiprazole 10 mg once daily. After approximately one year of follow-up, her depressive symptoms, anxiety, and irritability had remitted. No clinically significant adverse effects or weight changes were observed, and both medications were continued at these dosages.

Because no suitable in-person program was available, alternative educational options were explored collaboratively with her and her family. Through this process, she recently enrolled in a formal online upper-secondary education program completed from home, with daily assistance from her mother to organize activities, understand instructions, and complete academic tasks. Although this option allowed her to resume formal education, it offered fewer opportunities for in-person social and community participation than her previous educational and community settings. As the program had only recently begun, its longer-term educational, social, and emotional effects could not yet be evaluated.

She expressed sadness about being unable to return to the organizations she had previously attended because she had exceeded their age limits. She also expressed a desire to pursue a university degree and have a romantic partner, reflecting goals that extended beyond symptom management and basic daily functioning to include continued education, meaningful relationships, community participation, and greater autonomy. Her mother had devoted substantial time to supporting her since childhood and again became the principal source of educational, social, and practical assistance after formal services ended.

Case #2 on adult identification without a pathway forward

A 26-year-old man was evaluated by our team after two previous clinical evaluations, as difficulties became increasingly apparent during the transition to university and greater independence. Developmental history revealed no reported significant developmental delays or major developmental concerns. Throughout childhood and adolescence, however, he generally preferred interacting with one or two familiar people, showed little interest in larger-group activities, and maintained two close friendships. His communication style was typically brief, direct, and concrete; he rarely initiated conversation solely for social purposes or spontaneously elaborated on emotional experiences, although he could communicate his needs and respond appropriately when directly asked.

He maintained good academic performance throughout primary and secondary education. After entering university, however, he became increasingly overwhelmed by academic, organizational, and social demands and by the greater independence and flexibility expected of him. He withdrew after one semester.

Following his withdrawal, his daily activities and structured routine decreased substantially, and he developed depressive and anxiety symptoms, increased social withdrawal, and reduced motivation and participation in previous activities. Within approximately six months, he began psychotherapy, which continued for approximately two years with partial improvement. During this period, he was treated with fluoxetine 20 mg once daily and clonazepam 0.5 mg once daily for approximately two years. Following his second clinical evaluation, olanzapine 10 mg once daily was added and continued for approximately the final year of this period, after concern was raised for a possible prodromal phase of schizophrenia based primarily on increasing social withdrawal, reduced eye contact, and his direct and concrete communication style.

During subsequent psychiatric reassessment by our team, the previously raised concern regarding possible psychotic symptoms was specifically evaluated. Clinical interview and mental status examination identified no delusions, hallucinations, formal thought disorder, disorganized behavior, or other clinically significant psychotic features, and the available history did not indicate that such symptoms had occurred during the depressive episode. Reconstruction of his developmental history further indicated that his social-communication characteristics had been present since childhood and preceded the onset of depression.

Further assessment included clinical interview, developmental history, observation of current functioning, and psychometric measures of autistic characteristics. He obtained a score of 159 on the Ritvo Autism Asperger Diagnostic Scale-Revised (RAADS-R), 38/50 on the 50-item Autism-Spectrum Quotient (AQ), and a T-score of 66 on the Social Responsiveness Scale, Second Edition (SRS-2), corresponding to the moderate range of clinically significant social-communication difficulties. These findings were interpreted alongside his developmental history and clinical presentation rather than as stand-alone diagnostic measures. Taken together, the assessment supported identification as autistic in adulthood.

Fluoxetine, clonazepam, and olanzapine were gradually discontinued, and treatment was transitioned to venlafaxine 75 mg once daily and risperidone 1 mg once daily. Risperidone was subsequently discontinued, while venlafaxine 75 mg once daily has been maintained to the present. Approximately six months after this treatment modification, he had returned to a level of daily functioning similar to that observed before the onset of his depressive symptoms. At the one-year follow-up, he remained emotionally stable, with sustained remission of his previous depressive and anxiety symptoms. No psychotic symptoms emerged during follow-up.

As his emotional and functional status improved, an opportunity within his church community facilitated his return to structured daily activity and employment. Although initially intended to be temporary, the position continued, and he had been working at the church for approximately two years at the time of this report. An online university program had previously been considered by the individual and his family, but the emergence of mental health concerns shifted the immediate focus toward his recovery. With greater clinical and functional stability, educational alternatives were explored collaboratively with him and his family, including options for resuming university studies through an online program. However, at the time of this report, no formal pathway for educational re-entry or individualized adult support had been established. Written informed consent for publication of this case report was obtained from the individual.

## Discussion

Different pathways, yet the same discontinuity of support

The two individuals followed different pathways into adulthood but encountered a similar gap in coordinated support. In the first case, structured educational, therapeutic, and community supports available during childhood and adolescence ended when the individual exceeded their age limits. In the second, identification as autistic in adulthood provided a developmental framework for understanding longstanding experiences but was not followed by a formal pathway for educational re-entry or individualized adult support.

The shared issue was therefore not the complete absence of assistance but the lack of a coordinated pathway across adulthood. Both individuals eventually accessed alternatives that addressed important aspects of their lives: an online educational program supported by the family in Case #1 and employment within a familiar church community in Case #2. These experiences also demonstrate that useful support need not originate exclusively from healthcare or specialized autism services. Families, educational settings, employers, and community organizations may all contribute to participation when their involvement is appropriate to the individual’s needs and goals.

Continuity of support in this context should not be understood as continuous clinical care, indefinite supervision, or permanent dependence on services. Rather, it refers to maintaining access to appropriate and adaptable pathways when changes in needs, environments, or personal goals might otherwise disrupt continuity across adulthood. Transition planning can help identify these needs before age-limited services end and facilitate coordination across educational, healthcare, employment, and community settings [4,15]. Similarly, adults identified as autistic later in life may benefit from practical post-identification guidance that extends beyond diagnostic explanation and is responsive to their individual priorities [11,12].

Taken together, the cases illustrate that discontinuity may occur at different points: through the loss of established supports at the transition to adulthood or through the absence of a practical pathway following adult identification. The objective is not to maintain every previous service indefinitely but to avoid abrupt gaps and facilitate access to supports and opportunities that promote participation, self-determination, and autonomy.

Emotional stability does not equal inclusion

Both individuals experienced meaningful improvement in their depressive and anxiety symptoms with psychiatric care. Although this represented an important clinical outcome, educational, occupational, relational, and community barriers remained. Our team worked with both individuals and their families to identify feasible options beyond clinical care. In Case #1, this process supported her return to formal education through an online program, although suitable in-person educational and community opportunities remained limited. In Case #2, clinical improvement made a return to university feasible; however, limited access to inclusive educational environments and the absence of a structured pathway for a gradual, supported return remained important barriers to resuming in-person studies.

A report of two cases cannot determine whether the disruption or absence of support caused the emotional difficulties observed in either individual. Nevertheless, the temporal association between changes in routine, participation, and mental health highlights the importance of evaluating psychiatric symptoms within the broader context of the person’s environment. Autistic adults may experience barriers to care that extend beyond symptom recognition and treatment, including difficulties accessing services that are adapted to their communication needs, preferences, and life circumstances [16].

Clinical follow-up should therefore consider outcomes beyond symptom reduction alone. Relevant questions include whether the person has access to meaningful activities, whether educational or workplace demands are appropriately adjusted, whether opportunities for social and community participation are available, and whether support corresponds to the individual’s own priorities. Research on mental healthcare for autistic adults has similarly emphasized the need for services that bridge clinical treatment with practical, individualized support rather than ending once acute symptoms have improved [17].

Emotional stability may increase a person’s capacity to participate in education, employment, relationships, and community life, but it does not create accessible opportunities by itself. Meaningful inclusion depends on the interaction between the individual and the surrounding environment, including the availability of appropriate accommodations, supportive relationships, and opportunities for participation.

Adult identification should be the beginning of support

Adult autism identification can provide a meaningful framework for understanding longstanding experiences and may allow individuals to reinterpret previous social, educational, occupational, and mental health difficulties from a developmental perspective [11]. In the second case, identification as autistic helped provide a more coherent understanding of social-communication characteristics that had been present since childhood and adolescence and that had previously been interpreted, in part, within a psychiatric framework. However, diagnostic clarification alone could not address the limited availability of inclusive and adaptable educational environments or provide the structured support needed for a gradual return to university.

Autistic adults have described substantial variability in the information and assistance available following diagnosis, with particular gaps in practical and longer-term support [11-13]. Post-identification support may therefore be most useful when it moves beyond diagnostic explanation and helps the individual identify current needs, personal goals, existing strengths and resources, and potential barriers to participation.

Support after adult autism identification should be based on the person’s needs, circumstances, and goals. It may involve help with understanding the diagnosis, accessing appropriate mental healthcare, obtaining educational or workplace accommodations, exploring vocational or community opportunities, or navigating available services. Research suggests that autistic adults and their support persons particularly value assistance that is practical, accessible, and relevant to their everyday lives [14]. The need for such support varies between individuals and over time; adult autism identification does not, in itself, imply a need for continuous professional care. The type and intensity of support should reflect the individual’s own goals and may change over time.

The second case also demonstrates the potential contribution of resources outside formal healthcare. His church community provided an employment opportunity within a familiar environment and facilitated his return to structured activity. Community, educational, and employment settings may therefore complement clinical services by creating opportunities for participation that healthcare alone cannot provide. Adult identification should consequently be viewed not as an endpoint, but as a potential starting point for identifying which forms of support, accommodation, or community resources, if any, are meaningful to the individual as they pursue their own goals. 

Families and communities cannot carry the entire responsibility

Families played an important role in both cases. In Case #1, the mother searched for alternatives after age-limited services ended and provided the daily assistance needed for her daughter to participate in online education. In Case #2, family support extended across several stages, including helping him navigate his educational trajectory, facilitating his connection to a work opportunity within his community, and supporting the clinical evaluations that ultimately contributed to a clearer understanding of his needs. These examples illustrate how family involvement can provide continuity, practical knowledge, and individualized support across educational, occupational, and clinical transitions.

Family participation can be particularly valuable when it reflects the autistic person’s preferences and goals and is integrated into shared decision-making. However, access to education, healthcare, employment, and community participation should not depend exclusively on a family’s ability to identify, coordinate, or provide support. Families differ substantially in their available time, resources, knowledge, and capacity to navigate fragmented systems. Approaches to adult support should therefore recognize families as potential partners when desired by the autistic person, while also ensuring that appropriate opportunities remain accessible to individuals with limited or unavailable family support [2,18].

Community networks may similarly provide meaningful forms of support. In Case #2, his church community provided an employment opportunity within a familiar environment, facilitating a return to structured daily activity and participation. Community organizations, employers, educational institutions, and other naturally occurring networks may offer opportunities for belonging, skill development, social participation, and employment that formal healthcare services cannot provide. Such resources should therefore be recognized as potentially important components of adult support rather than viewed merely as substitutes for specialized services [10,19].

At the same time, meaningful participation should not depend on belonging to a particular community or having personal contacts able to create an opportunity. Community-based and informal supports are most equitable when they complement accessible educational, employment, healthcare, and social resources rather than becoming the only available pathway. A flexible approach that combines formal and naturally occurring supports according to the individual’s preferences and circumstances may reduce gaps while promoting participation and autonomy rather than dependence.

Education, employment, and relationships are interconnected

Education, employment, relationships, and community participation are closely interconnected in adult life. Education may contribute not only to learning and future employment but also to social participation and identity development; employment can provide income, structure, opportunities to develop skills, and connection with the broader community; and friendships and intimate relationships can foster belonging, emotional well-being, and self-determination. Opportunities and barriers in one domain may therefore influence experiences and possibilities in others [2,4].

Employment outcomes among autistic adults are heterogeneous, and obtaining a job does not necessarily mean that employment-related needs or aspirations have been fully addressed. Research has identified challenges in obtaining and maintaining employment while also emphasizing the importance of job fit, workplace accommodations, individual preferences, and appropriate support [20-23]. In Case #2, an opportunity within his church community developed into sustained employment, which he had maintained for approximately two years at the time of this report. This represents a meaningful outcome in its own right rather than a temporary substitute for formal services. At the same time, employment does not replace his educational aspirations, and he continues to consider returning to university.

Both cases illustrate the importance of flexible and individualized educational pathways. Online education may provide a viable option for some autistic adults by reducing specific environmental or organizational barriers, but it should be considered alongside opportunities for autonomy, relationships, vocational development, and community participation rather than as a substitute for them [2]. In Case #1, online education enabled a return to formal study with daily assistance from her mother, although opportunities for in-person social and community participation remained limited. In Case #2, online university education is being considered as one possible route for resuming his studies, while a gradual return to a supportive in-person environment may also be feasible. Educational planning should therefore reflect individual circumstances, preferences, goals, and support needs rather than assuming that a single format is appropriate for all autistic adults.

Relationships should likewise be recognized as a legitimate and meaningful domain of adult life. The first individual’s desire for a romantic partner is not a clinical problem to be treated but a personal aspiration deserving the same consideration as educational or employment goals. Autistic adults may encounter barriers to friendship, intimacy, and relationship-related education, including access to appropriate information about sexuality, consent, and personal safety [24]. When consistent with the individual’s preferences, adult support may therefore extend beyond mental health care, daily functioning, education, and employment to include opportunities for friendship, intimacy, community involvement, and self-determination.

Taken together, these cases support an approach in which successful adult outcomes are not defined by conformity to a predetermined model of independence but by the autistic person’s own preferences, goals, circumstances, and support needs. The purpose of support is not to normalize behavior or eliminate reliance on others, but to reduce barriers and expand meaningful opportunities for participation and autonomy.

Implications for practice and service organization

These cases highlight the need for clearer and more adaptable pathways across major transitions in adult life. Before school-based or age-limited services end, transition planning should identify educational, occupational, social, and autonomy-related goals; determine which existing supports should continue or be modified; and identify accommodations or alternative resources that may be needed as circumstances change [2,4,15].

A similar process may be useful following autism identification in adulthood. Beyond providing diagnostic information, clinicians should offer an opportunity to discuss current priorities, support needs, and available resources. Depending on the individual, this may involve mental healthcare, educational or workplace accommodations, vocational guidance, peer support, or community resources, with the level of assistance adjusted over time according to the person’s preferences and circumstances [11,12,14].

Such pathways do not need to depend exclusively on specialized autism or healthcare services. When desired by the autistic person, families may provide valuable knowledge and practical assistance, while educational institutions, employers, religious organizations, and other community networks can contribute opportunities and resources beyond the scope of clinical care [2,10,18]. In employment, appropriate workplace accommodations and individualized support may facilitate participation and job sustainability when aligned with the person’s abilities, preferences, and goals [22]. Coordination among clinical, educational, occupational, family, and community resources may therefore be more practical and sustainable than relying on any single system.

Psychiatric care remains important when depression, anxiety, or other co-occurring mental health concerns affect well-being or functioning, but symptom improvement does not necessarily remove barriers in other areas of adult life. Mental healthcare may therefore be most useful when it is accessible and individualized while remaining connected, when appropriate, to relevant educational, occupational, and community supports [16,17]. The goal is not continuous care or indefinite supervision, but continuity of access as needs and goals evolve. Successful transitions should therefore be evaluated not only through symptom remission, service use, employment, or independence, but also through progress toward personally meaningful goals, reduced environmental barriers, and opportunities for education, relationships, community participation, and autonomy.

## Conclusions

These two individuals followed different pathways into adulthood but encountered a similar challenge: the absence of clear and adaptable pathways as their needs and circumstances changed. One lost established educational and community supports after exceeding the age limits of childhood services. The other gained a more coherent understanding of longstanding experiences after being identified as autistic in adulthood, yet lacked a structured pathway for a gradual and supported return to university. In both cases, families played a central role, while collaborative efforts with the clinical team and available community resources helped identify feasible alternatives. However, these efforts could not fully compensate for the limited availability of coordinated and inclusive adult supports. Clinical improvement in co-occurring mental health symptoms is an important outcome, but it does not by itself constitute inclusion or a successful transition to adulthood. Support should remain individualized and adaptable, facilitating access to personally meaningful opportunities in education, employment, relationships, community participation, and autonomy as goals and circumstances evolve. Responsibility for navigating what comes next should therefore not rest primarily on autistic people and their families but be shared across healthcare, educational, employment, social, and community systems.
